# Illicit Drug Use and Problem Gambling

**DOI:** 10.1155/2013/342392

**Published:** 2013-08-25

**Authors:** Peter Ferentzy, W. J. Wayne Skinner, Flora I. Matheson

**Affiliations:** ^1^Centre for Addiction and Mental Health, 33 Russell Street, Toronto, ON, Canada M5S 2S1; ^2^Department of Psychiatry, University of Toronto, 8th Floor, 250 College Street, Toronto, ON, Canada M5T 1R8; ^3^Faculty of Social Work, University of Toronto, 246 Bloor Street West, Toronto, ON, Canada M5S 1V4; ^4^Centre for Research on Inner City Health, The Keenan Research Centre, Li Ka Shing Knowledge Institute of St. Michael's Hospital, 30 Bond Street, Toronto, ON, Canada M5B 1W8; ^5^Institute for Clinical Evaluative Sciences, Room G1 06, 2075 Bayview Avenue, Toronto, ON, Canada M4N 3M5; ^6^Dalla Lana School of Public Health, University of Toronto, Health Science Building, 6th Floor, 155 College Street, Toronto, ON, Canada M5T 3M7

## Abstract

Problem gambling, substance use disorders, and their cooccurrence are serious public health concerns. We conducted a comprehensive review of the literature to understand the present state of the evidence on these coaddictions. Our main focus was illicit drug use rather than misuse of legal substances. The review covers issues related to gambling as a hidden problem in the illicit drug use community; prevalence, problem gambling, and substance use disorders as kindred afflictions; problem gambling as an addiction similar to illicit drug use; risk factors and problems associated with comorbidity, and gender issues. We end with some suggestions for future research.

## 1. Introduction

The relation between illicit drug abuse and problem gambling remains understudied, with a preponderance of information generated from treatment samples. Research on problem gambling itself suggests stark differences between gamblers seeking treatment and those identified in the general population: treatment populations are generally white, middle-aged men while those in the general population are more likely than treatment populations to be women, minorities, and of lower education [1–3]. Moreover, a sizable number of problem gamblers and substance abusers have mental health problems, particularly antisocial personality disorder, which may inhibit treatment participation [4]. Conversely, the so-called “Berkson bias” would suggest that, with most conditions, cooccurrence of disorders increases the likelihood that someone will seek treatment [5, 6]. While informative in its own right, information gathered from treatment samples remains limited. 

A meta-analysis of available prevalence studies of gambling was conducted by the Division on Addictions at Harvard Medical School [7]. This was a landmark meta-analysis of 152 studies conducted between 1977 and 1997, including 35 Canadian studies. Findings showed that over the previous 25 years, the estimated prevalence of gambling problems in the general adult population had been low but rising. The estimated lifetime prevalence in the general adult population for problem and pathological gambling combined was reported at 6.72% for more recent studies (conducted between 1994 and 1997) in comparison to a mean prevalence of 4.38% among older studies (conducted between 1977 and 1993). There were no significant differences in prevalence rates between the United States of America and Canada. A more recent review [8] showed that the prevalence of past year excessive gambling varied with a low of 0.6% to a high of 6.4%.

Canadian statistics on drug use are available through the 2009 Canadian Alcohol and Drug Use Monitoring Survey. Recent reports revealed that cocaine or crack (1.2%) was, after Cannabis, the illicit drug most commonly used in the past 12 months, followed by ecstasy (0.9%), hallucinogens (0.7%), speed (0.4%), and methamphetamine (0.1%). The prevalence of use of at least one of six drugs (Cannabis, cocaine or crack, speed, ecstasy, hallucinogens, or heroin) in the past year was 11.0%. The rate of use by males (14.7%) was almost double that by females 7.6%, and the prevalence of use was more than three times higher among youth (27.3%) than adults (7.9%). Use of at least one of five illicit drugs excluding Cannabis (cocaine or crack, speed, ecstasy, hallucinogens or heroin) was reported by 2.0% of Canadians. The reported rate of such use by males (2.5%) was not statistically different from that reported by females (1.5%). However, the rate of use by youth (5.5%) was almost four times higher than that reported by adults at 1.3% [9]. 

The literature from the 1990s shows how awareness of cooccurring problem gambling and substance use disorder was just emerging. Griffiths [10] found that substance use disorder treatment workers seemed to consider illicit drug addiction to be all-consuming and, hence, to believe that drug addicts have no time, energy, or money left to gamble. Shepherd [11] wrote specifically on obstacles to administering the South Oaks Gambling Screen (SOGS) to patients at a methadone and alcoholism clinic and reported facing noncompliance by staff who considered gambling irrelevant or at least as secondary to substance addiction. Of note was a seeming belief among staff that the purchase of lottery tickets by persons with low incomes could not be a major problem, even if the behavior consumed substantial portions of total income. 

Despite limitations to current knowledge, high correlations between problem gambling and substance use disorders have been widely reported [12–16]. Steinberg et al. [17] found that 15% of cocaine abusers under study were also pathological gamblers, ten times the rate found in community samples. In their review of the literature, Spunt et al. [2] found that problem gambling rates among those who abuse substances (alcohol and other drugs) are four to ten times that of the general population. Boas de Carvalho et al. [18] found high problem gambling rates (10.8% problem and 18.9% pathological) among 74 (89% male) persons in treatment for substance use disorders (of whom 60.3% identified cocaine/crack as a major drug of abuse). In a community sample, El-Guebaly et al. [19] found persons with substance use disorders exhibit problem gambling at a rate 2.9 times that of persons with no identified disorder. Within a substance use treatment sample, Langenbucher et al. [20] found that 13% met problem gambling criteria. Spunt [21] found that out of 462 New York City methadone patients, 21% were probable pathological gamblers and another 9% were problem gamblers. Crockford and El-Guebaly [4] reviewed the literature on psychiatric comorbidity in pathological gambling and found in both community and clinical samples that between 25% and 63% of pathological gamblers met the criteria for a substance use disorder.

Evidence suggests that problem gamblers with substance use disorders might be less receptive to current treatment initiatives [22]. Spunt et al. [2] also report that problem gamblers in methadone treatment settings received little support or sympathy from peers. Though possibly dated, the study by Grodsky and Kogan [23] suggests that gambling is also perceived by professionals as a hidden problem—one that clients are more likely to deny than substance abuse—and greater acceptance of medical rather than moral conceptions of substance use disorder is identified as a reason. Given the marginal and essentially hidden nature of the target population—street drug users with gambling problems—unique approaches are necessary to encourage engagement of participants in research [24].

In a review of the literature, Griffiths et al. [25] made five pertinent observations about the relationship between problem gambling and substance abuse: (1) some types of gambling may be more likely than others to cooccur with substance use disorders (slot machines are offered as a possibility); (2) given that problem gambling is multifaceted, probably a syndrome rather than a single disorder, this will have implications for treatment among those with comorbidities; for instance, problem gamblers with and without certain substance use disorders may differ in motivation to seek and persist in treatment; (3) gender, culture, and age are related in complex ways with drugs of choice, as well as with gambling and substance use patterns; (4) both substance abuse and problem gambling have a temporal dimension, and researchers interested in understanding the comorbidity of problem gambling and substance abuse will need to consider different stages of gambling and substance use careers, and (5) while problem gambling researchers tend to be well aware of the significance of comorbid substance use disorders, those in the substance use disorder field rarely give problem gambling the same consideration.

It has long been understood that problem gambling and substance use disorder are kindred afflictions [26, 27], and the current Diagnostic Statistical Manual designation of problem gambling as a disorder of impulse control does not alter the fact that the identified symptoms are modelled on substance use disorders [28–32]. Spunt [21] discusses similarities between pathological gambling and substance abuse, pointing to the common symptoms such as loss of control, disease progression, and even tolerance—it is common for pathological gamblers to require increasingly large bets in order to achieve the desired level of excitement and for drug users to develop tolerance requiring greater frequency, and higher doses to achieve a high ([25, 28], see also [33–35]). 

Petry [31] points out that both problem gambling and substance use disorders typically begin in adolescence or early adulthood, that each condition is known to wax and wane, and that natural recovery seems common to both afflictions (see also [12, 31, 36–41]). Boughton and Falenchuk [42] mention the relatively small number of people with problem gambling (4%) who ever seek treatment, which in turn speaks to the importance of self-change in the recovery process. Unassisted recovery is known to be common with both problem gambling and substance use disorders [43, 44]. The latter development, not very well understood even as it plays out in more mainstream settings, is obviously a bigger question mark in settings that are hidden, marginal, and alienated from many facets of mainstream authority (including that of researchers). 

It has been argued that problem gambling and substance use disorders are related because of underlying traits such as impulsivity [45–47], adding more weight to the notion of a unified disorder. Still, such a conception is unlikely to apply to every case [25] since most information on comorbidity comes from treatment samples we may have exaggerated the associations given that persons with more than one disorder might be more inclined to seek help [12]. Crockford and El-Guebaly [4] suggest that since gambling and substance use often occur at the same locations, some cases of comorbidity may be situational rather than endemic to the disorders. 

## 2. Designations: Addiction, Impulse Control Disorder, and Dependence

Petry [31] discusses the pros and cons of reclassifying and broadening the substance use disorders to include nonpharmacological addictions such as pathological gambling. Points in favour of expansion to other addictive disorders—in this case problem gambling—include high rates of comorbidity, similar symptoms, and demographics, as well as physiological and genetic commonalities ([31], see also [48]). Points against reclassification include the fact that with problem gambling no substance is ingested. Moreover, substance abusers often experience relief after withdrawal symptoms subside, but problem gambling leaves long-term financial issues in its wake. As well, some phenomena do not translate. Substance use disorder has no real parallel for chasing losses, and problem gambling has no real parallel for many drug induced health hazards [31]. Longterm illicit drug use can entail serious chronic health conditions that are not apparent among problem gamblers [31, 49].

Making a case for an excessive appetite model of addiction, Orford [50] offers examples of appetitive activities that can qualify as addictive behaviors such as drinking, gambling, sex, and eating. Only behaviors with skewed consumption distribution curves qualify. In short, certain behaviors are clearly engaged in excess by a small minority and, often, the patterns of distribution are remarkably similar for different addictive behaviors (see also [51]). One of Orford's [50] major arguments is that over-specified conceptions of maladaptive behaviors can lead to neglect of social determinants associated with a range of behaviors.

Impulse control disorder holds a poorly defined place in the larger body of psychiatric disorders [32, 52]. Conditions that many would consider addictions, such as kleptomania and pyromania, are currently classified as International Classification of Diseases [28]. Given that problem gambling and substance use disorders are often marked by underlying impulsivity, a case could be made for treating the two as elements of a single disorder [31, 46, 53]. Rosenthal [27] has pointed out that all addictions are, by necessity, disorders of impulse control (see also [54, 55]). 

Just as problem gambling's International Classification of Diseases designation is based upon a substance use disorder/addiction model, the current designation of substance use disorder as “dependence” has been described as an addiction model [56]. The two markers many would associate with physiological “dependence”—tolerance and withdrawal—are included as possible symptoms but, on their own, are neither necessary nor sufficient to obtain a “dependence” diagnosis. Other symptoms, such as loss of control and social or economic dysfunction, are required [28]. It is expected that the next Diagnostic Statistical Manual may well identify both problem gambling and substance use disorder as addictions [28, 57]. 

## 3. Risk Factors for Comorbid Problem Gambling and Substance Use Disorders

There is a substantial body of literature on risk factors that are associated with both problem gambling and substance use disorders. In an adolescent study, Winters and Anderson [58] noted that the likelihood of gambling involvement increased with drug use, and that the risk factors for drug use and problem gambling overlap significantly with predictable markers such as sexual abuse, depression, and delinquency.

Studies of adolescents lend weight to the idea that problem gambling and substance use disorders are in many cases functions of an underlying behavioral disorder (problem behavior syndrome and conduct disorder), predictably marked by factors such as impulsivity, low parental supervision, and deviant friends [59]. Among youth, such factors can be predictive of behaviors ranging from substance use and gambling to theft and violence [60]. Barnes et al. [61] have discussed these matters, and offer moral disengagement as another explanatory construct. While useful—and especially so for youth studies and prevention initiatives—these overreaching conceptions do not account for how, among adults with either problem gambling or illicit drug addiction, most criminal behaviors do not precede but seem rather to stem from addiction [62, 63]. 

Slutske et al. [64] studied 4,497 male twin pairs to assess the association between problem gambling and antisocial personality disorder (ASPD). Suggesting the presence of a genetic link, they argue that antisocial personality disorder should not necessarily be regarded as simply an offshoot of problem gambling. These authors claim that problem gambling may be more strongly associated with antisocial personality disorder than with any other psychiatric disorder, including substance use disorder. Evidence for this line of thought, however, has been inconsistent. Crockford and El-Guebaly [4] argue that, while a sizable minority of people with problem gambling have antisocial personality disorder, most seem to develop antisocial traits as a result of gambling. With substance use disorders, evidence suggests that antisocial personality disorder is but one out of four main psychiatric correlates, the others being borderline, avoidant, and paranoid personality disorder [65].

Among psychiatric risk factors, impulsivity is perhaps the best predictor for comorbid problem gambling and substance use disorders [46, 66, 67]. Noting that evidence for impulsivity among problem gamblers is less consistent than for those with substance use disorders, Petry [47] suggests that high substance abuse rates among gamblers can help to explain this inconsistency.

Clarke et al. [48] identify several key indicators for the development of both problem gambling and substance abuse including low economic status, reduction of negative affect, loss of control, problem-conducive environments, glamorous advertising, and social inducements. In a study of female gamblers (74% probable problem gamblers), Boughton and Falenchuk [42] reported high rates of physical and emotional abuse and speculated that problem gambling may often be a substitute for behaviors ranging from substance abuse to binge eating and compulsive shopping. In a sample of primarily African-American low-income and out-of-treatment female substance abusers, Cunningham-Williams et al. [68] found that antisocial personality disorder was significantly associated with the increased likelihood of problem gambling, primarily driven by items relating to violent tendencies (particularly the intersection of drug use and fighting behavior). Crockford and El-Guebaly [4] noted high rates of cooccurring problem gambling and substance use disorders among Native Americans, suggesting that issues such as ethnicity and culture need to be considered. These authors also note that the settings in which pathological gambling and substance abuse occur are often the same. This obviously makes it difficult to sift out questions concerning availability of drugs and gambling as predictors from other factors such as abuse, criminal contacts, and deprivation (see, e.g., [69]). 

## 4. Problems and Behaviors Associated with Comorbidity

Cooccurrence of problem gambling and substance use disorders is associated with more personal and social difficulties [70, 71]. Liu et al. [72] found that out of 1390 past year recreational gamblers, 142 (10%) with substance abuse problems gambled more heavily, had started to gamble at earlier ages, and were more likely to gamble in the hopes of winning money. These authors speculated that financial problems associated with substance use disorders may account for a stronger desire to win. Problem gambling has also been associated with increased substance use among adolescents [73]. 

Kausch [74] found that persons with both problem gambling and substance use disorder histories were more likely to have attempted suicide at some point in their lives and to report problems with sexual compulsivity. Spunt [21] found gambling to be a potential reinforcer of drug use, an obstacle to success in treatment, and also a substitute activity that may become exacerbated upon abstinence from drugs. In a five-year-follow-up study of problem gamblers who had quit gambling, Hodgins and El-Guebaly [75] reported that persons with comorbid drug use histories were less likely to achieve short-term abstinence from gambling. Weinstock et al. [76] found that methadone maintenance patients with problem gambling had poorer mental and physical health than those without gambling problems. McCormick [77] found that polysubstance abusers were more likely to exhibit serious problem gambling than those with only one substance addiction.

Ciarrocchi [78] found that chemically dependent problem gamblers reported more chronic medical problems, more suicide attempts, and more conflicts with relatives and family members than nonchemically dependent psychiatric patients. Ladd and Petry [46] found that among problem gamblers admitted to treatment, those with substance use disorder histories had more severe problems on the gambling, alcohol, drug, psychiatric, and employment scales of the Addiction Severity Index. Langenbucher et al. [20] found that persons with substance use disorders who were also problem gamblers scored higher on measures of severity of substance use disorders as well as psychiatric comorbidity. Ledgerwood and Downey [79] found that methadone patients with probable problem gambling were more likely to use cocaine during treatment and to drop out of the clinic treatment program, while Toneatto et al. [80] reported that drug use history did not interfere with problem gambling treatment.

Persons with substance use disorders may also use gambling to support their drug habits [21, 24, 72]. Spunt et al. [3] found that those with substance use disorders were more likely to use substances before or while gambling to enhance performance and also to assuage the pain of losing and enhance the joy of winning. Seemingly, one attraction of drugs is the predictability of pleasure, as opposed to the uncertainty associated with gambling. 

Substance use disorders could be a major indicator of impulsivity in many problem gambling cases. Petry [81] found that problem gambling among persons with substance use disorders was further associated with risky sexual conduct. Possibly, we are dealing with an underlying impulse control disorder [47, 81], though impulse control disorder is arguably a catchall with an ambiguous place in the larger schema of psychiatric disorders [32, 52]. Either way, a range of psychiatric indicators, including obsessive compulsive disorder, paranoia, hostility, sexual disinhibition, anxiety, negative affect, and poor coping, has been associated with comorbid problem gambling and substance use disorders [45, 69, 78, 81–83]. In a study of willingness to delay small rewards in favour of larger rewards to come later, Petry and Casarella [84] found that persons with substance use disorders forsook delayed rewards at higher rates than controls, and that persons with substance use disorders in addition to problem gambling did so at even higher rates. 

Substitution is a potential concern with problem gambling and substance use disorder [21, 42, 74, 85, 86]. Given that both problem gambling and substance use disorders are known to wax and wane [87], this is understandable. It seems that in the absence of illegal drug purchasing, and the many behaviors that go with it, gambling can provide an alternative form of excitement [3]. Current research suggests that in most cases a substance use disorder precedes problem gambling [4, 88], so gambling's role as an alternative is an important consideration. Crockford and El-Guebaly [4] stress caution around substitution. In their review, they identified two studies that found a limited degree of symptom substitution and one study that did not [89, 90]. Moreover, only one study looked at the natural histories of pathological gambling and substance use disorders, with the most common pattern being that the onset of the substance use predates the onset of the gambling problems [4, 91]. Alternatively, Carnes et al. [86] discussed the idea of cross-tolerance, specifically that the need for greater stimulation due to tolerance within one area (problem gambling or substance use) could increase tolerance for extremes in another. As well, for each condition the timing of onset is often later with women and disease progression is more rapid—a phenomenon referred to as telescoping [67, 92].

Vadhan et al. [93] provide a good example of just how uncharted some of this subject matter really is. There has been a decent amount of evidence for cognitive impairment caused by the use and overuse of psychoactive substances [94–98]. Yet Vadhan et al. [93] found that cocaine smokers displayed none of the expected cognitive dysfunctions on the Iowa Gambling Task when payment for study participation was geared to performance. This was admittedly a small study, but these authors legitimately question whether some (though obviously not all) of the short-term cognitive impairment long considered to be caused by cocaine abuse might instead be a motivational issue. Two points surface immediately. First, given that illicit drug users are marginalised and at times misunderstood, it is possible that certain biases have led the research community to overstate negative traits (in this case cognitive impairment). Second, since the cognitive distortions associated with pathological gambling mainly involve delusions specific to gambling, notably regarding the odds of winning or losing [31], the identification of cognitive dysfunction specific to comorbid problem gambling and substance use disorder is laden with unknowns. If nothing else, the study by Vadhan et al. [93] suggest that, when properly motivated, cocaine smokers can gamble as efficiently as anyone else. 

The idea of substitution of disorders is an emerging field, with authors such as Widyanto and Griffiths [99] discussing the potential difficulty in simply identifying what someone may be addicted to when, say, they gamble or act out sexually on the Internet. Efforts to determine precisely what substance use disorders and problem gambling behaviors do for certain youths, for example, pose similar difficulties and have led some to invoke problem behavior syndrome [59, 100]. Impulsivity [32] is a helpful idea and possibly related to a penchant for risk taking that is also associated with problem gambling, substance use disorders, and other maladaptive behaviors [101–103]. Research on substitution issues related to comorbid problem gambling and substance use disorders might have to look past the targeted behaviors and, possibly, address substitution as an issue in itself.

## 5. Gender, Problem Gambling, and Substance Use Disorders

Males have long been overrepresented in problem gambling study samples [3, 19, 53, 87, 104–106]. The ratio of men to women among problem gamblers has been estimated at between 2 : 1 and 3 : 1 [7, 107–109]. Yet with changes in the availability and legal status of many gambling activities, it would seem that the number of women with problem gambling is on the rise. Evidence for this was already surfacing over ten years ago [1, 2, 110]. More recently, in an Australian sample Crisp et al. [111] found that out of 1,520 persons seeking help for gambling related difficulties, 46% were female. There is also good evidence that Gamblers Anonymous, once an almost exclusively male organization, is now host to more women with each passing year [112]. 

Prevalence rates for illicit substance use have typically been estimated as higher among men than women [113–118]. Yet we are dealing with many substances, some of which are more strongly associated with misuse and addiction than others. While available evidence does favour an overall gender imbalance for use of harder drugs [114, 115, 117], reliable information that is also more detailed has been harder to generate. In one study of crack users, women reported more crack use, though men were more likely to use injection drugs [119]. In a study of opiate addicts, women were more likely to use crack in conjunction with heroin, while men were more likely to use benzodiazepines [120]. In a discussion of the more recently popular practice of injecting crack itself, Clatts et al. [121] identify difficulties in attempting to quantify changing behaviors among these hidden populations, and also observe that patterns of episodic use (which often seems to be the case with crack injection) are even harder to gauge. Clearly, gender ratios pertaining to many drug using and related behaviors are hard to ascertain.

It is at least possible that gender differences in problem gambling rates are not as stark among persons with substance use disorders as they are among the general population. In a study of 465 New York City methadone patients, Spunt et al. [3] found that 25% of the men and 15% of the women were probable problem gamblers. In a treatment seeking sample, Toneatto and Brennan [122] found the rates to be 11.9% for men and 7.5% for women. Since we are dealing with a hidden or hard to reach population, confidence in current estimates of illicit drug use and problem gambling within demographic categories (still based primarily on treatment samples) would be premature [2]. 

Roles and behaviors are embedded in gender socialization patterns. Males are generally more likely to sell drugs, and females are more likely to sell sex in order to obtain drugs [123, 124]. Spunt et al. [3] found that both male and female problem gamblers in methadone treatment continued to gamble, but that males were more likely to commit crimes and to engage in various hustling behaviors related to gambling. Males were more likely to participate in a wider variety of gambling activities, while females were more likely to play bingo and pull tabs (see also [19]). While males and females in this sample were very similar in patterns of drug use and choice of substances while gambling, more males than females reported using heroin while gambling. Among men and women who reported using heroin while gambling, men identified heroin as a means to enhance concentration while women pointed to increased confidence when high [3].

Some findings have arguably been unsurprising. In a study of substance use patterns in a problem gambling treatment sample, Toneatto and Skinner [116] found that gender differences in overall drug use were proportionally consistent with such differences found in the general population, with women using more legal medications, for example, and men using more illicit drugs (though rates of use and abuse of illicit drugs were higher for each gender in this sample than in the general population). 

Possibly, of greater significance there has been evidence regarding the so-called telescoping effect: as with substance use disorders, the time of onset for problem gambling in women is often at later ages but then the disorder typically progresses more rapidly [32, 67, 125, 126]. Desai and Potenza [92] argue that this can, at least in part, be explained by reference to underlying psychiatric issues. These authors attempted to gauge comorbid psychiatric disorders among persons with subclinical gambling problems, specifically to determine whether these disorders were more prominent among women than men with gambling problems. The results were positive, and though the study involved subclinical problem gamblers, rates of eight psychiatric comorbidity indicators [28] increased in step with increases in problem gambling severity among women.

In a study of 78 problem gambling patients (36 female and 42 male), Dannon et al. [66] found that men had higher rates of substance use disorders, whereas women scored higher on depression, anxiety, and eating disorders. While explanations and interpretations must still be considered preliminary, these authors hypothesize that male and female problem gamblers may have different types of impulsive behavior, with men primarily failing to resist internal, harmful urges and women failing to resist egodystonic cognitions and obsessive thoughts [66]. In a youth study, Nower et al. [127] found that stress-related coping strategies distinguished nongamblers from problem gamblers. For instance, male nongamblers were more likely to adopt task-oriented coping strategies; in comparison male problem gamblers avoided stressors by engaging in distracting behaviors/activities and/or through use of fantasy, substance use and denial. Among females, coping strategies were not significantly associated with gambling, but again female nongamblers were more likely to use task-oriented coping strategies and less likely to engage in substance abuse than females who were problem gamblers [127]. 

Matheson et al. [128] asked men and women to describe the negative consequences and positive aspects of gambling and how this is related to seeking help for, and recovery from, problem gambling. Men reported that the emotional response to the addiction (i.e., shame, anxiety, sense of failure, depression, and anger) was a major obstacle to recovery, while for women it was the seductiveness of the gambling environment (e.g., resolve your financial problems, acquire large sums of money quickly, and receive perks from casinos). Ellenbogen et al. [129] suggest that gender differences found among adult problem gamblers figure already during adolescence, with young female gamblers, for example, more likely to report gambling as an escape from difficulties rather than as a competitive activity (see also [12]).

Evidence has long suggested that women respond differently to substance use treatment than men [130, 131]. Treatment programs often recognize the necessity of a more holistic approach for women with behavioral problems. For example, a program to treat substance use, and other behavioral problems associated with criminality was developed for federally sentenced women offenders. This program recognizes the need to address early trauma that can lead to criminality and substance abuse including childhood sexual and physical abuse [132]. Similar findings have surfaced for problem gambling. Lesieur [133] claimed that discussing a range of compulsions (rather than simply the target addiction) matters more to women gamblers. Crisp et al. [134] found male gamblers to report employment and legal matters as important, whereas with women physical and interpersonal issues surfaced as key. Boughton and Falenchuk [42] have discussed how many women opt for self-help manuals in order to address their gambling problems, and Crisp et al. [111] interpret their own results as suggesting that women are more amenable to community based, nonresidential approaches to counselling. Arguably, these findings suggest that women likely require more supportive counselling, possibly psychotherapy, whereas men prefer information sharing and cognitive restructuring [126]. 

## 6. Directions for Future Research

One of our goals was to provide a review of the literature on the affinities between problem gambling and substance use disorders. What became apparent when we delved into the literature on this topic is the pervasiveness of social marginalization. We are dealing with a complex-needs population with substance use disorders and concurrent mental illness, including personality disorders; all ingredients in a recipe for poverty, social exclusion, homelessness, violence, prostitution, and chronic illness. There is a substantial body of literature on risk factors that are associated with both problem gambling and substance use disorders; including sexual abuse, depression adolescent delinquency and impulsivity. Research is needed to explore how men and women experience this social marginalization. What are the consequences—social, emotional, and health—of such a marginal status in society? 

Our review also showed that the majority of studies on problem gambling and substance abuse draw on treatment samples which are relatively easy to access. But, findings from the smaller number of studies with nontreatment populations suggest important differences in those who seek treatment and those who do not. Treatment samples differ in demographics (e.g., gender, ethnicity, and socioeconomic status) from nontreatment samples. Those seeking treatment may also differ in their experience of comorbid disorders; those with multiple problems might be more willing to enter treatment. Research exploring differences between the treatment and nontreatment populations might help us to identify new approaches to reach out to those who shy away from treatment. 

Vadhan et al. [93] have questioned whether certain types of drug use affect short-term cognitive impairment. Given the potential biases of researchers in such matters, these assumptions ought, at the very least, to be revisited. While drug use is usually viewed as a problem, Erickson et al. [123, page 775] suggested that it may also represent a potential solution for persons in dire need. Despite the problematic nature of addictive behaviors, more attention needs to be given to what draws people to harmful behaviors such as problem gambling and substance use disorders. 

Substitution of one behavior for another has long been identified as a key issue in the field of addictions, and perhaps the very notion of substitution could be studied in its own right. It could very well be that in some cases, the primary addiction is not to any one behavior but to a process where the object can be and does get replaced and alternated.

Erickson et al. [123] observed that many on the fringes of society cannot even imagine themselves as part of the mainstream. This reminds us that the purpose of knowing about patterns of behavior such as illicit drug use and gambling is less to save people from the brink and more to accurately understand how life is lived on the margins; to empathically hear what the concerns, needs, and goals of people living there are; and to advance a deepened awareness of what leads people to the margins, how life is conducted there, and how we can enhance the choices they have available to them. 
